# Tumors induce de novo steroid biosynthesis in T cells to evade immunity

**DOI:** 10.1038/s41467-020-17339-6

**Published:** 2020-07-17

**Authors:** Bidesh Mahata, Jhuma Pramanik, Louise van der Weyden, Krzysztof Polanski, Gozde Kar, Angela Riedel, Xi Chen, Nuno A. Fonseca, Kousik Kundu, Lia S. Campos, Edward Ryder, Graham Duddy, Izabela Walczak, Klaus Okkenhaug, David J. Adams, Jacqueline D. Shields, Sarah A. Teichmann

**Affiliations:** 1 Department of Pathology, University of Cambridge, Cambridge, CB2 1QP UK; 2 Wellcome Sanger Institute, Wellcome Genome Campus, Hinxton, Cambridge, CB10 1SA UK; 3 EMBL-European Bioinformatics Institute, Wellcome Genome Campus, Hinxton, Cambridge, CB10 1SD UK; 4 Medical Research Council Cancer Unit, Hutchison/Medical Research Council Research Centre, Cambridge, UK; 5 Department of Biology, Southern University of Science and Technology, Shenzhen, China; 6 Department of Haematology, University of Cambridge, Cambridge Biomedical Campus, Long Road, Cambridge, CB2 0PT UK; 7 Theory of Condensed Matter, Cavendish Laboratory, 19 JJ Thomson Ave, Cambridge, CB3 0HE UK; 8 Present Address: Translational Medicine, Research and Early Development, Oncology R&D, AstraZeneca, Cambridge, United Kingdom

**Keywords:** Cancer microenvironment, Tumour immunology

## Abstract

Tumors subvert immune cell function to evade immune responses, yet the complex mechanisms driving immune evasion remain poorly understood. Here we show that tumors induce de novo steroidogenesis in T lymphocytes to evade anti-tumor immunity. Using a transgenic steroidogenesis-reporter mouse line we identify and characterize de novo steroidogenic immune cells, defining the global gene expression identity of these steroid-producing immune cells and gene regulatory networks by using single-cell transcriptomics. Genetic ablation of T cell steroidogenesis restricts primary tumor growth and metastatic dissemination in mouse models. Steroidogenic T cells dysregulate anti-tumor immunity, and inhibition of the steroidogenesis pathway is sufficient to restore anti-tumor immunity. This study demonstrates T cell de novo steroidogenesis as a mechanism of anti-tumor immunosuppression and a potential druggable target.

## Abstract (web summary)

Multiple mechanisms of immune evasion exploited by cancer cells have been described. Here, the authors show that genetic inactivation or pharmacological inhibition of tumor-induced Th2-mediated de novo steroidogenesis are sufficient to restore an efficient anti-tumor immune response and restrict tumor growth.

## Introduction

**Fig. 1:**
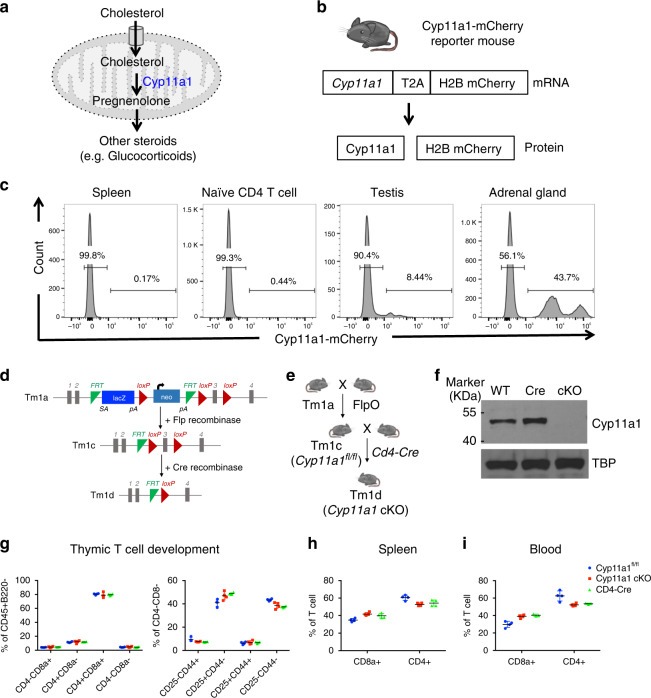
Generation of a de novo steroidogenesis reporter and conditional knockout mice. **a**. Schematic of de novo steroidogenesis pathway.  Cyp11a1 is the first and a key rate-limiting enzyme of the pathway. **b**. *Cyp11a1*-reporter mice synthesize a fusion protein that self-cleaves due to presence of a T2A peptide and dissociates into Cyp11a1 and H2B-mCherry. **c** Cyp11a1-mCherry reporter mice report Cyp11a1 expression accurately. Single-cell suspensions of tissues and naïve splenic CD4^+^ T cells were analyzed by flow cytometry. Gating: All cells>Singlets>Live cells>Cyp11a1-mCherry. Representative of three independent experiments; each experiment contains 3–4 mice. **d**, **e** Generation of a Cyp11a1 conditional knockout (cKO) mice. Schematic presentation of the The targeting allele (**d**) and T cell-specific Cyp11a1 cKO (*Cd4*^−^Cre;*Cyp11a1*^fl/fl^) generation (**e**). **f** Cyp11a1 knockout efficiency of Cre recombinase in T cells. Splenic naïve T helper cells from cKO (*Cd4*-Cre;*Cyp11a1*^fl/fl^) mice or control mice (wild type and *Cd4*-Cre) were activated under Th2 differentiation condition, and analyzed for Cyp11a1 protein expression by western blot. TATA-binding protein (TBP) used as loading control. **g** Normal thymic development of T cells in Cyp11a1 cKO. Thymus was harvested from Cyp11a1 cKO and control (*Cd4*-Cre and *Cyp11a1*^fl/fl^) mice, dissociated into single-cell suspension, stained with fluorescent conjugated anti-CD4, CD8, B220, CD45, CD25, and CD44 antibodies, and analyzed by flow cytometry. Gating: all cells>Singlets>Live cells>CD45^+^B220^−^>CD4, CD8 (left panel). CD4^+^CD8^+^ cells represent double positive (DP) stage, CD4^+^CD8^−^ and CD4^−^CD8^+^ cells represent single positive (SP) stage, CD4^−^CD8^−^ cells represent double negative (DN) stage. DN cells of the left panel were gated to show CD25 and CD44 expression to identify DN1 (CD25^−^CD44^+^), DN2 (CD25^+^CD44^+^), DN3 (CD25^+^CD44^−^), and DN4 (CD25^−^CD44^−^). Error bars represent mean with s.d., *N* = 4 biologically independent animals. (**h**, **i**). Flow cytometric analysis shows normal distribution of Cyp11a1 cKO T cells (CD4^+^ and CD8^+^) in the peripheral blood and spleen. Error bars represent mean with s.d., *N* = 4 biologically independent animals.

Steroidogenesis is a metabolic process by which cholesterol is converted to steroids^1^. The biosynthesis of steroids starting from cholesterol is often termed de novo steroidogenesis^1^. Cytoplasmic cholesterol is transported into the mitochondria, where the rate-limiting enzyme CYP11A1 (also known as P450 side chain cleavage enzyme) converts cholesterol to pregnenolone. Pregnenolone is the first bioactive steroid of the pathway, and the precursor of all other steroids (Fig. 1a)^1,2^. The steroidogenesis pathway has been extensively studied in adrenal gland, gonads, and placenta. De novo steroidogenesis by other tissues, known as extra-glandular steroidogenesis, in brain^1,3^, skin^4^, thymus^5^, and adipose tissues^6^ has also been reported. Steroid production as a result of immune response in the mucosal tissues, such as in the lung and intestine, has been shown to play a tolerogenic role to maintain tissue homeostasis^7,8^. However the physiological and pathological role of extra-glandular steroidogenesis remains largely unknown^2^.

In cancer, the immunosuppressive tumor microenviroenmnt (TME) prevents immune cells from mounting an effective anti-tumor immune response^9^. Several mechanisms by which cancer cells evade the immune system have been described^10,11^. These include: (1) immune suppression at the TME mediated by immunosuppressive cells, (2) induction of apoptosis in cytotoxic T-lymphocytes (CTLs) by the expression of pro-apoptotic ligands e.g., Fas ligand and TRAIL, (3) dysregulating antigen presentation, (4) release of immunosuppressive factors such as IL-10 and TGFβ, and (5) inducing tolerance and immune deviation by mechanisms including, among others, shifting the balance of Th1 immune responses (type-1 immune response) to Th2 (type-2 immune response), and expression of immune inhibitory molecules such as PD-1 (programmed death-1) and CTLA-4 (CTL antigen-4). Established tumor often show type 2 immune responses that drive an immunocompromised and pro-tumorigenic program^12–25^. Further understanding of the anti-tumor immunosuppression would allow us to develop immunotherapies.

Steroid hormones are known immunosuppressive biomolecules^26,27^. We recently reported that type 2 CD4^+^ T cells (Th2 lymphocytes) induce de novo steroidogenesis to restore immune homeostasis by limiting the immune response against a worm parasite^28^. Thus we sought to determine whether type 2 T cell-mediated steroidogenesis contributes to the generation of a suppressive niche in the TME.

To study de novo steroidogenesis we generated two transgenic mouse lines: a fluorescent reporter mouse line (*Cyp11a1*-mCherry) and a conditional *Cyp11a1* (floxed) knockout mouse line. We show the presence of de novo steroidogenesis by tumor-infiltrating T lymphocytes, but not in unchallenged animals or draining lymph nodes. Genetic ablation of *Cyp11a1* in T cells restricts experimental primary tumor growth and lung metastasis. Mechanistically, we find that intratumoral T cell steroidogenesis dysregulates anti-tumor immunity that could be restored by inhibiting the steroidogenesis pathway pharmacologically. This study therefore demonstrates that T cell de novo setroidogenesis is a cause of anti-tumor immunosuppression and a potential drug target for cancer immunotherapy.

## Results

### Generation of *Cyp11a1* reporter and conditional knockout mice

Cyp11a1 is the first and rate-limiting enzyme during steroid production. The expression of *Cyp11a1* is therefore also a faithful biomarker of de novo steroidogenesis^1^. Therefore, we generated a reporter mouse line to identify Cyp11a1-expressing steroidogenic cells definitively (Fig. 1b, c, Supplementary Fig. 1a–d). As expected, mCherry expression was detected in single-cell suspensions of testis and adrenal glands but negligible to no expression in the spleen (Fig. 1c) or other tissues including lung, kidney, blood, liver, bone marrow, lymph nodes, and  thymus (Supplementary Fig. 1b). However, Cyp11a1-mCherry signal was detected specifically in activated type-2 CD4^+^ T helper cells (Th2 cells) upon activation in vitro (Supplementary Fig. 1c), as reported previously^28^. Cyp11a1 expression was detectable only in mCherry-expressing T helper cells (Supplementary Fig. 1d).

To determine the functional consequences of cell-type-specific steroidogenesis we created a *Cyp11a1* floxed (*Cyp11a1*^*fl/fl*^) mouse following EUCOMM/WSI conditional gene targeting strategy^29^. Briefly, a knockout-first (tm1a) mouse line was created using a promoter-driven targeting cassette (Fig. 1d). The *tm1a* mouse was then crossed with Flp-deleter mice (FlpO) to remove the *LacZ* and *Neo* cassette, and generate a *tm1c* allele (i.e. *Cyp11a1*^*fl/fl*^). When crossed with a Cre-driver, the Crerecombinase removes exon 3 of *Cyp11a1* gene and creates a frameshift mutation (Fig. 1d). Because we had initially detected Cyp11a1 expression in Th2 cells^28^, we crossed the *Cyp11a1*^*fl/fl*^ line with a *Cd4*-driven Crerecombinase to delete *Cyp11a1* and prevent de novo steroidogenesis in all T cells (Fig. 1e). Deletion efficiency of Crerecombinase in the *Cyp11a1* cKO (*Cd4-Cre;Cyp11a1*^*fl/fl*^) mice was nearly complete in Th2 cells (Fig. 1f). *Cyp11a1* cKO mice showed normal thymic development of T cells, and a normal distribution in the peripheral tissues (Fig. 1g–i).

### In vitro analysis of Cyp11a1 expression in T cells

**Fig. 2:**
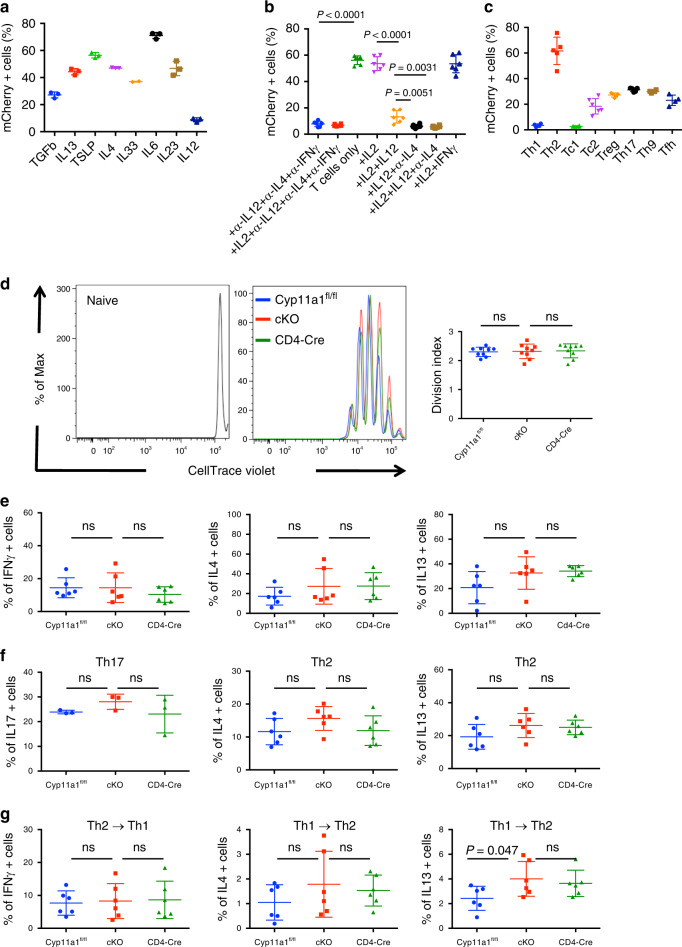
Analysis of Cyp11a1-expressing T cells using Cyp11a1 reporter and cKO mice. **a** Splenic naïve CD4^+^ T cells from *Cyp11a1*-mCherry reporter mice were purified by negative selection; activated in the anti-CD3e/anti-CD28 antibody coated plates in the presence of cytokines for 3 days, rested for 2 days, restimulated 6 h, and Cyp11a1-mCherry expression was analyzed by flow cytometry. *N* = 3 (except IL33 where *N* = 2). **b** IL12 inhibits Cyp11a1 expression. Splenic naïve CD4^+^ T cells from Cyp11a1-mCherry reporter mice were activated as mentioned above (**a**) for 5 days and Cyp11a1-mCherry expression was analyzed by flow cytometry. *N* = 6. **c** Splenic naïve CD4^+^ and CD8^+^ T cells from Cyp11a1-mCherry reporter mice were activated in vitro under Th1, Th2, Th9, Th17, Tfh, Treg, Tc1, and Tc2 differentiation conditions (activation 3 days, resting 2 days), and Cyp11a1-mCherry expression was analyzed by flow cytometry. *N* = 6 (Tc1, Tc2), *N* = 4 (Th1, Th9, Tfh), and *N* = 5 (Treg, Th17). **d** Splenic naïve CD4^+^ T cells were purified from *Cyp11a1* cKO, *Cd4*-Cre, and *Cyp11a1*^fl/fl^ mice, stained with CellTrace Violet, activated in vitro, and cell proliferation was determined by a flow cytometric dye decay assay. Representative cell proliferation profiles are shown in the left panel and a comparison of the cell division index is shown in right panel. *N* = 9. **e** Splenic naïve CD4^+^ T cells were activated in vitro in the absence of any exogenous cytokine or cytokine-neutralizing antibody, and cytokine expression was determined by flow cytometry. *N* = 6. **f** Splenic naïve CD4^+^ T cells were activated in vitro under Th17 or Th2 differentiation conditions, and cell type-specific signature cytokine expression was determined by flow cytometry. *N* = 3 (Th17), *N* = 6 (Th2). **g** Splenic naïve CD4^+^ T cells were activated in vitro under Th1 or Th2 differentiation condition. After 3 days Th1 cells were allowed to differentiate under Th2-polarizing conditions (Th1 > Th2), and Th2 cells were allowed to differentiate under Th1-polarizing conditions (Th2 > Th1). Th1 or Th2-specific cytokine expression was determined by flow cytometry. *N* = 6. All error bars in this figure represent mean with s.d. *P*-value was calculated using unpaired two-tailed *t*-test. Representative of three independent experiments. *N* represents biologically independent animals.

Exploiting our *Cyp11a1*-mCherry reporter line, we assayed a panel of cytokines commonly found in inflammatory settings, including tumors, for their ability to induce steroidogenesis in CD4^+^ T cells. IL4, IL6, IL13, and TSLP induced a strong *Cyp11a1*-mCherry signal in in vitro activated CD4^+^ T cells (Fig. 2a, Supplementary Fig. 2a, b). In contrast, IL12 had an inhibitory effect on *Cyp11a1*-mCherry expression (Fig. 2b, Supplementary Fig. 2c). This indicated that not only Th2 lymphocytes, but also other T cell types, are capable of de novo steroidogenesis, at least in vitro. To test this, we differentiated naïve CD4^+^ or CD8^+^ T cells into Th1, Th2, Th9, Th17, Tfh, Treg, Tc1, and Tc2 subsets in vitro. All subsets examined, with the exception of Th1 and Tc1 cells, exhibited *Cyp11a1*-mCherry expression when activated, consistent with an inhibitory role for IL12 in Cyp11a1 regulation (Fig. 2c, Supplementary Fig. 2d). These data suggest that Cyp11a1 expression, and thereby steroidogenesis, is a default process during T cell activation, and is inhibited by the presence of IL12. Overall, of all T cell subtypes generated in vitro, Th2 cells exhibited the highest percentage of Cyp11a1 expression and Th1 cells do not express Cyp11a1.

To determine the requirement of Cyp11a1 activity for T helper cell proliferation and differentiation, we purified naïve splenic T cells from *Cyp11a1* cKO and control mice. We activated the cells in vitro to generate different subclasses of T helper cells, and analyzed signature cytokine expression by flow cytometry. In the absence of Cyp11a1, T cells proliferate normally (Fig. 2d). Cyp11a1 expression was not required for the differentiation of any T helper cell type tested as determined by signature cytokine expression (Fig. 2e, f, Supplementary Fig. 2e). We observed that deletion of *Cyp11a1* in T cells does not interfere with the plasticity of T helper cells (Fig. 2g, Supplementary Fig. 2f, g).

As a next step, Cyp11a1 induction in T cells  was investigated in vivo.

### Tumors induce functional Cyp11a1 expression in T cells

Tumor-infiltrating T cells are key fate determinants within a tumor, but are often suppressed^30^. The steroidogenesis-inducing type-2 cytokines such as IL4 are also often present in the TME^31,32^, thus we next sought to examine the steroidogenic capacity (i.e. Cyp11a1 induction) of T cells infiltrating tumors, and their impact on tumor development. To explore Cyp11a1 expression in vivo, we utilized the well-established B16-F10 melanoma model^33–35^ and implanted tumors subcutaneously in *Cyp11a1*-mCherry reporter mice.

**Fig. 3:**
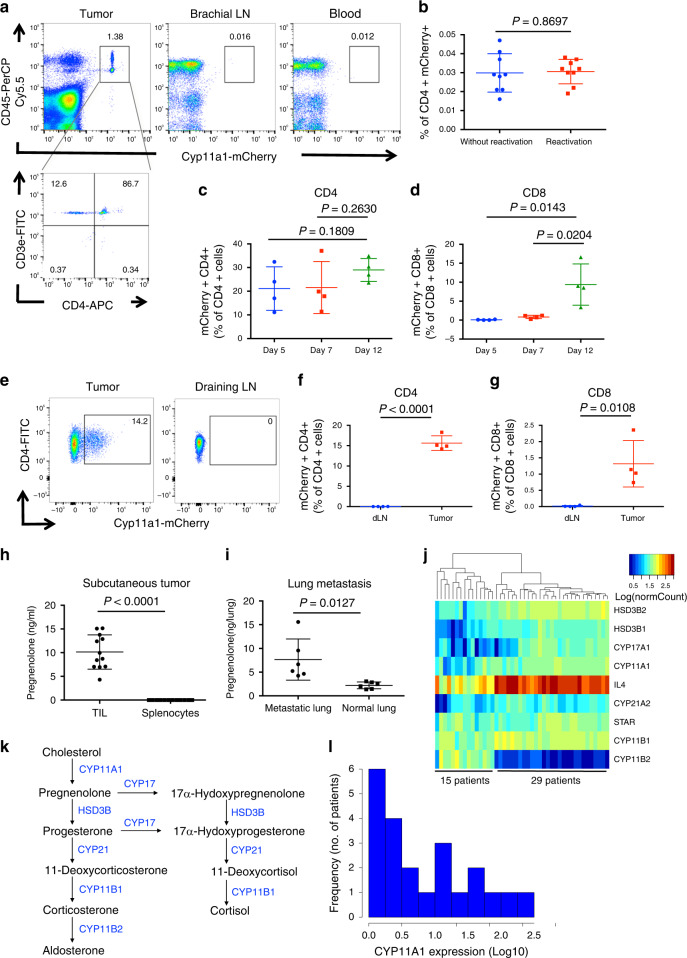
Tumors induce Cyp11a1 expression in T cells in vivo. **a** B16-F10 cells were injected subcutaneously into the *Cyp11a1*-reporter mice. After 12 days brachial lymph node (LN), blood, and tumor tissues were analyzed by flow cytometry. Gating strategy: Singlets>Live cells>CD45,Cyp11a1-mCherry. *N* = 5. CD45^+^Cyp11a1-mCherry^+^ cells were further gated to show T helper cell (CD4^+^CD3e^+^) expression of Cyp11a1. **b** Splenic cells were purified from tumor-bearing *Cyp11a1*-reporter mice, and restimulated in vitro using PMA/ionomycin and analyzed by flow cytometry. *N* = 9. **c**, **d** B16-F10 cells were injected subcutaneously into the Cyp11a1-mCherry reporter mice. After 5, 7, and 12 days tumor tissues were analyzed by flow cytometry to detect the CD4^+^ T cells (**c**), CD8^+^ T cells (**d**) *N* = 4. All cell>Singlets>Live cell>CD45^+^>CD4^+^CD3e^+^ or CD8^+^CD3e^+^. **e**–**g** EO771 cells were injected into the mammary fat pad of *Cyp11a1*-reporter mice. After 15 days, tumor tissues and tumor-draining LN were analyzed by flow cytometry to detect the Cyp11a1-mCherry expression in CD4^+^ T cells (**e**, **f**) CD8^+^ T cells (**g**). Gating: All cell>Singlets>Live cell>CD45^+^>CD4^+^TCRb^+^ or CD8^+^TCRb^+^. **e** Representative FACS profile of Cyp11a1^+^CD4^+^ T cells. **f**, **g** Representative graphical presentation of one experiment showing Cyp11a1-expressing CD4 and CD8 T cells. *N* = 4. **h** B16-F10 tumor-infiltrating leukocytes (TIL) and splenocytes were purified from tumor-bearing mice on post-inoculation day-12, cultured for 48 h, and the supernatant was analyzed by ELISA to measure pregnenolone. *N* = 12, pooled analysis of three independent experiments. **i** Metastasized lungs were harvested 10 days post-B16-F10 intravenous injection in C57BL/6 mice, dissociated into single-cell suspension, cultured for 48 h, and the supernatant was analyzed by ELISA. Naïve uninfected lungs (normal lung) were used as control. *N* = 6, pooled analysis of two independent experiments. **j** Hierarchical clustering of steroidogenic genes and *IL4* mRNA expression across 44 melanoma patient samples (GEO: GSE19234). **k** Schematics showing glucocorticoid synthesis pathway. **l** Frequency distribution histogram showing *CYP11A1* mRNA expression (normalized read counts, log10 scale) across 22 melanoma patients’ tumor-infiltrating CD4^+^ T cells (EGAD00001000325). Individual data points for *CYP11A1* expression are shown in Supplementary Fig. 3i. All error bars in this figure represent mean with s.d. *P*-values were calculated by unpaired two-tailed *t*-test. *N* represents biologically independent animals.

Cyp11a1 expression was detected in immune cells of established primary tumors, but not in tumor-draining brachial lymph nodes (LN) or blood (Fig. 3a), indicating that stimulation occurs in situ. In support of this, stimulation of splenic T cells did not induce Cyp11a1 expression ex vivo (Fig. 3b, Supplementary Fig. 3a). The dominant Cyp11a1^+^ tumor-infiltrating immune cells were identified as T cells, predominantly CD4^+^ (helper T cells, Fig. 3a).

Having identified presence of Cyp11a1-expressing T cells in established tumors, we next examined expression dynamics during tumor progression. Using the *Cyp11a1*-mCherry reporter mice we observed expression of Cyp11a1 in CD4^+^ T cells which remained stable during development of the tumor through days 7 and 12. By contrast, CD8^+^ T cells only expressed Cyp11a1 by day 12 (Fig. 3c, d). Expression dynamics of minor and rare Cyp11a1^+^ non-T cells (mast cells and basophils) is shown in Supplementary Fig. 3b, c.

To determine whether Cyp11a1 induction in T cells is conserved in other tumor types we analyzed an EO771 orthotopic model of breast cancer^36–38^ (Fig. 3e–g, Supplementary Fig. 3d). Again, we found that Cyp11a1 was upregulated in tumor-infiltrating T cells (Fig. 3e–g), but not in tumor-draining lymph node or spleen. We also detected minor and rare populations of other Cyp11a1^+^ type-2 immune cells, such as mast cells and basophils (Supplementary Fig. 3d).

Next, we sought to measure the functional output of Cyp11a1 expression. Significant concentrations of the steroid pregnenolone were detected exclusively in immune cells isolated from tumors, with negligible levels detected in cells from the spleen (Fig. 3h). Using the B16-F10 model of experimental metastatic dissemination^39^, we determined that lungs with metastatic nodules, but not control lungs without metastatic nodules, had elevated levels of pregnenolone (Fig. 3i).

The presence of tumor cells did not induce or augment the Cyp11a1 expression in T cells in vitro (Supplementary Fig. 3e). This indicates that type-2 (Th2) cytokines within the TME induce Cyp11a1 expression by T cells and this cannot readily be mimicked in vitro.

Having observed steroidogenic T cells in murine melanoma, we turned to publicly available transcriptomic data sets to verify our findings and ascertain relevance in the human setting. We identified *CYP11A1* mRNA expression, and thus steroidogenic potential, in a range of cancer types including liver, breast, prostate, lung, kidney, sarcoma, glioma, uterine, cervical, lymphoma, and melanoma (Supplementary Fig. 3f, g)^32^. Human melanoma tissues represented a prominent steroidogenic tumor type, expressing *CYP11A1, HSD3B1, HSD3B2, CYP17A1, CYP21A1*, and *CYP11B1* and not expressing *CYP11B2* (Fig. 3j, k). CYP11B2 catalyzes the aldosterone synthesis from corticosteroid precursor (Fig. 3k). Together this was indicative of melanoma-driven production of glucocorticoids (Fig. 3k, Supplementary Fig. 3h).

Interestingly, in melanoma, steroidogenic gene expression was correlated with *IL4* expression (Fig. 3k, Supplementary Fig. 3j), a key inducer of T cell steroidogenesis^28^. Moreover, analysis of human tumor-infiltrating CD4^+^ T cell transcriptomes, confirmed *CYP11A1* expression (Fig. 3l) implying that CD4^+^ T cells are a source of steroids in tumors, mirroring the murine setting. Collectively these data indicate that in both human and mice, TILs produce steroids within the tumor.

### Gene expression identity of Cyp11a1^+^ immunocytes in melanoma

**Fig. 4:**
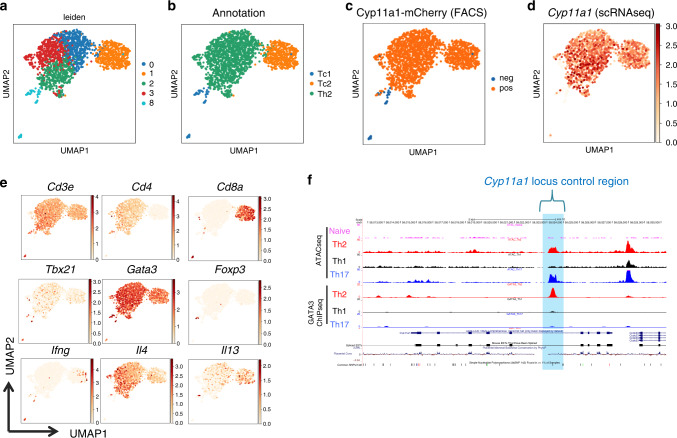
Revealing gene expression identity of intratumoral Cyp11a1^+^ T cells by scRNAseq. **a**, **b** UMAP visualization of the tumor-infiltrating T cells (**a**) with annotations of the clusters (**b**). Tc1 represents type-1 CD8^+^ T cells, Tc2 represents type-2 CD8^+^ T cells, and Th2 represents type-2 CD4^+^ T cells. **c**, **d** mCherry protein expression accurately reports *Cyp11a1* mRNA expression. Cyp11a1-mCherry protein expression according to the flow cytometry (FACS) data (**c**). *Cyp11a1* mRNA expression in the scRNA-seq data (**d**). **e** Expression of cell-type-specific signature genes that were used to annotate the clusters. **f** Determination of *Cyp11a1* locus control region. Open chromatin regions were identified by ATAC-seq of T helper cells. In vitro generated Th2 and Th17 cells were used as positive control. Naïve and Th1 cells were used as negative control. Gata3 ChIP-seq data were analyzed to determine the binding site. The open chromatin region where Gata3 occupy is highlighted blue and considered as locus control region.

To reveal the gene expression identity of intratumoral steroidogenic immune cells and patterns of gene expression at single-cell resolution, we inoculated B16-F10 subcutaneous tumor in *Cyp11a1*-mCherry reporter mice, enriched and purified intratumoral Cyp11a1-mCherry^+^ and Cyp11a1-mCherry^−^ cells by cell sorting into 96-well plates [with a ratio of 79:15 (mCherry^+^: mCherry^−^) cells per plate] and performed single-cell RNA sequencing (scRNA-seq) using the SMART-Seq2 platform^40^. The majority of cells, ~3000, passed quality control. Unsupervised hierarchical clustering and visualization with reduced dimensionality using UMAP (uniform manifold approximation and projection)^41^ identified 12 clusters of cells (Supplementary Fig. 4a). There was no batch effect between samples (Supplementary Fig. 4b) or impact of sex (Supplementary Fig. 4c) on clustering, and Cyp11a1-mCherry protein expression accurately reported *Cyp11a1* mRNA expression (Supplementary Fig. 4d, e). We annotated the clusters based on classical lineage markers (Supplementary Fig. 4a, f) and their signature gene expression (Supplementary Fig. 4g). The majority of the Cyp11a1-expressing cells are T cells as suggested by *Cd3e* expression (Supplementary Fig. 4f). T cells constitute the clusters 0, 1, 2, 3, and 8 with cluster-1 displaying genes of CD8^+^ Tc2 cells; *Cd8a, Cd3e*, *Gata3, and* type 2 cytokines *Il4, Il5, and Il13*. Clusters 0, 2, and 3 were CD4^+^ T helper cells (*Cd4*^+^ *Cd3e*^+^) as they expressed *Gata3, Il4*, and *Il13* (Supplementary Fig. 4f). As T cells were the dominant Cyp11a1-expressing population, we reanalyzed the data taking only the T cells and displayed in Fig. 4a–e. The three clusters of Th2 cells represent three closely related subpopulations (Fig. 4a, b, Supplementary Fig. 4a). The comparative gene expression between these clusters is shown in Supplementary Fig. 4i. In Supplementary Fig. 4a, Cluster-4 represents myeloid derived cells, macrophages, and dendritic cells, but mostly they constitute Cyp11a1-mCherry^−^ cells, though a very small number of Cyp11a1-mCherry^+^ cells have been found in this cluster. Cluster-5 contains B16-F10 cells and melanocytes from the host mice, and they were Cyp11a1^−^. Cluster-6 and 9 constitute two different states of the Cyp11a1^+^ mast cells, because they express *Kit* (the gene that encodes cKit*)* and *Fcer1* and other mast-cell-specific proteases such as *Mcpt4* and *Cma1*. The detailed differences between these two clusters have been shown in Supplementary Fig. 4j. Cyp11a1^+^ basophils and eosinophils (cluster-7) fall in the same cluster. Cluster-8 is a mixed population of CD4^+^ and CD8^+^ T cells. The number of cells in these two clusters are not sufficient to make a conclusion of their identity. Clusters 4, 5, 8, 11, and 10 are constituted by both Cyp11a1-mCherry^+^ and Cyp11a1-mCherry^−^ cells. Cyp11a1-mCherry^−^ cells representing the cells that are most frequently available cells within B16-F10 tumor, namely B16-F10 cells and macrophages.

Taking *Cyp11a1*-correlated genes we ran pySCENIC to identify potential transcription factors regulating *Cyp11a1* expression and found that the GATA family of transcription factors (Gata1, 2, 3) were predicted to be the controller of the *Cyp11a1* locus in these steroidogenic immune cells (Supplementary Fig. 4h). Gata3 is the lineage-defining TF of Th2 cells. To validate the prediction that Gata3 controls *Cyp11a1* expression in T helper cells, first we performed ATAC-seq to identify the open chromatin and potential locus control region (Fig. 4f). Next, we analyzed Gata3 ChIP-Seq data to confirm that Gata3 is indeed a potential regulator of the *Cyp11a1* locus in Th2 cells (Fig. 4f). The putative locus control region (blue highlighted region in Fig. 4f) was found in a closed-chromatin state in naïve and Th1 cells but was open in Th2 and Th17 cells and was occupied by Gata3 in Th2 cells (Fig. 4f).

All the Cyp11a1-expressing T cells express steroidogenic type-2 cytokines *Il4 and Il13* (Fig. 4e, Supplementary Fig. 4f). T cells express the cognate receptor for IL4 (*Il4ra*) indicating that these cells are receptive to the steroidogenic signal (Supplementary Fig. 4f).

The prediction of glucocorticoids in the tumor prompted us to check the cognate receptors in the tumor-infiltrating immune cells. We found that the glucocorticoid receptor *Nr3c1* was expressed in all tumor-infiltrating immune cells (Supplementary Fig. 4k).

### Ablation of T cell steroidogenesis restricts tumor growth

**Fig. 5:**
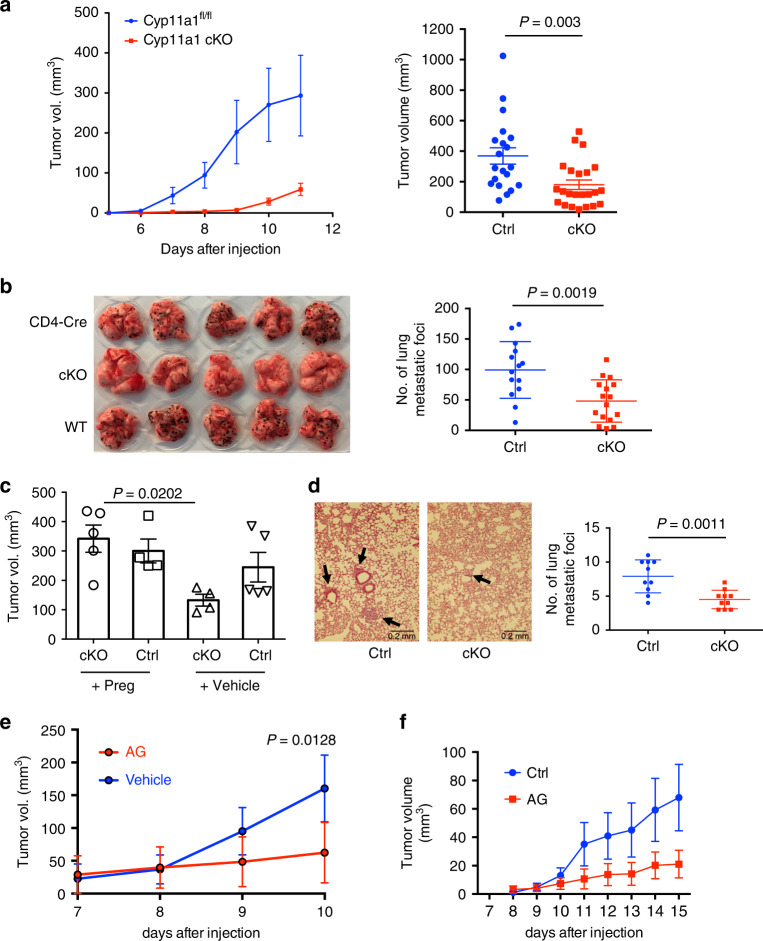
Ablation of T cell steroidogenesis restricts experimental tumor growth and metastasis. **a** Left-panel: B16-F10 subcutaneous tumor growth curve assessed in T cell-specific *Cyp11a1* cKO (cKO) and *Cd4*-Cre control mice. *N* = 5. Representative of four independent experiments. Right panel: graphical presentation of end-point tumor volume. *N* = 20 (ctrl), 23 (cKO). Pooled data of four independent experiments. Each point represents an individual animal. Error bars represent mean ± s.e.m., unpaired two-tailed *t*-test. **b** Left panel: Representative photograph of pulmonary metastatic foci produced 10 days after tail vein injection of B16-F10 cells in cKO and control (Cre and WT) mice. *N* = 5. Right panel: graphical presentation of the numbers of lung metastatic foci. *N* = 14 (ctrl), 16 (cKO), pooled data of three independent experiments, each point represents an individual animal, error bars represent mean with s.d. Unpaired two-tailed *t*-test. **c** cKO and *Cd4*-Cre control mice were injected with B16-F10 cells. Pregnenolone or vehicle (DMSO) applied topically at the primary tumor site every 48 h. Tumor volume was measured at the end-point at day 12. *N* = 5. Each point represents an individual animal. Error bars represent mean ± s.e.m. *P*-value was calculated by one-way ANOVA with Tukey Post hoc test. **d** Representative hematoxylin and eosin stained histologic photograph of pulmonary metastatic foci produced 10 days after tail vein injection of EO771 cells in *Cyp11a1* cKO and *Cyp11a1*^fl/fl^ mice. Graphical presentation of the numbers of lung metastatic foci (right panel). *N* = 10. Error bars represents mean with s.d. Unpaired two-tailed *t*-test. Representative of two independent experiments. **e** B16-F10 cells were injected subcutaneously in C57BL/6 mice with or without Cyp11a1 inhibitor aminoglutethimide (AG). AG treatment was continued with a 48-h interval. *N* = 5. Error bars represent mean ± s.e.m. Two-way ANOVA. Representative of two independent experiments. **f** EO771 cells were injected into the mammary fat pad in C57BL/6 mice with or without Cyp11a1 inhibitor AG. AG treatment was continued with a 48-h interval. *N* = 5. Error bars represent mean ± s.e.m. One experiment. In this figure, *N* represents biologically independent animals.

To determine the functional consequences of T cell-driven steroidogenesis in tumors we subcutaneously implanted *Cyp11a1* cKO mice with B16-F10 cells. Genetic deletion of *Cyp11a1* in T cells significantly restricted primary tumor growth rates and final volumes (Fig. 5a). Similarly, in the experimental metastasis model, impaired lung colonization was observed in the absence of T cell-expressed Cyp11a1. There was a significant reduction in number of metastatic foci in lungs of *Cyp11a1* cKO mice compared to the control mice (Fig. 5b). In the B16-F10 subcutaneous melanoma model, topical application of exogenous pregnenolone at the primary tumor site was sufficient to compensate for the Cyp11a1 deficiency, restoring tumor growth to levels comparable with control mice (Fig. 5c). This suggests that the tumor restriction was a consequence of the absence of pregnenolone synthesis. Similar results were obtained using the EO771 breast cancer orthotopic tumor model: deletion of *Cyp11a1* in T cells restricted dissemination to and nodule formation in the lung (Fig. 5d). Importantly, in both tumor models, pharmacological inhibition of Cyp11a1 by aminoglutethimide (AG) was sufficient to recapitulate the tumor restriction phenotype resulting from *Cyp11a1* genetic ablation (Fig. 5e, f). Together, these data indicate that T-cell-derived steroids can support tumor growth and both genetic and pharmacological interference with the pathway can restrict tumor growth.

### Inhibition of T cell steroidogenesis boosts tumor immunity

**Fig. 6:**
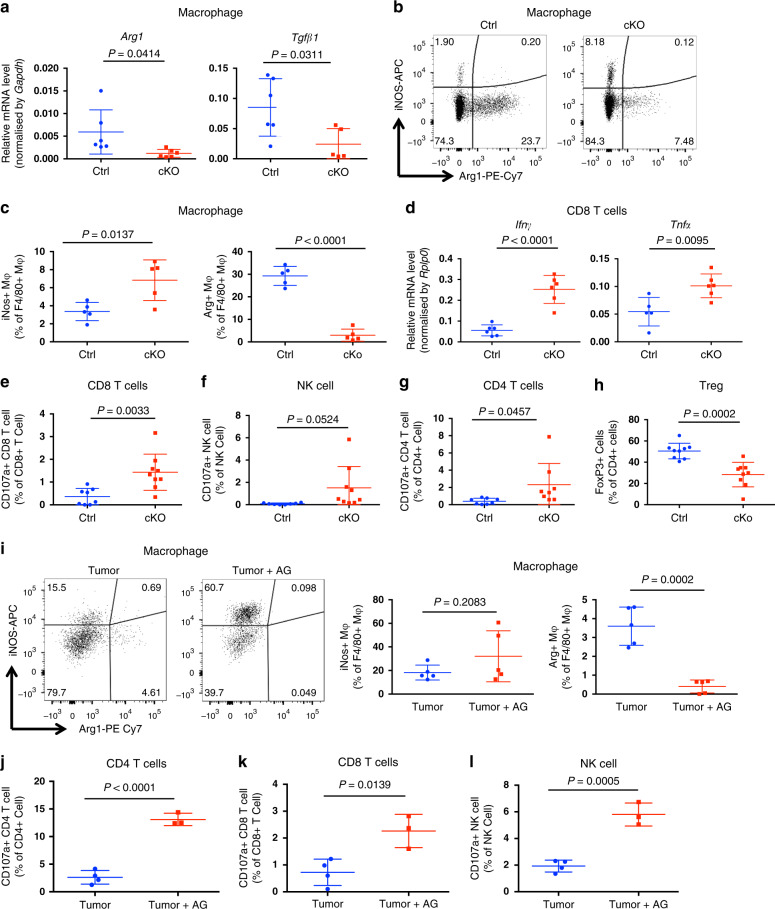
Inhibition of T cell steroidogenesis stimulates anti-tumor immunity. **a**–**h** Comparing *Cyp11a1* cKO and *Cd4*-Cre control mice with B16-F10 cells injected subcutaneously. Representative of three independent experiments. **a** Tumor-infiltrating macrophages (Lin^−^CD11b^+^) were purified by cell sorting at day 12. *Arg1* and *Tgfβ1* mRNA expression was quantified by RT-qPCR, with mRNA expression level normalized by *Gapdh* mRNA expression. *N* = 6. **b**, **c** Tumor-infiltrating macrophages (Lin^−^CD11b^+^F4/80^+^) analyzed by flow cytometry at day 12 to examine iNOS and Arg1 expression. Representative FACS profile of the expression (**b**). Representative graphical representation of one experiment (**c**). *N* = 5. **d** Tumor-infiltrating CD3e^+^CD8^+^ T cells purified by cell sorting at day 12, reactivated ex vivo, and *Ifnγ* and *Tnf*α mRNA expression quantified by RT-qPCR, with mRNA expression level normalized by *Rplp0* expression. *N* = 6. **e** Cytotoxic T-lymphocyte degranulation assay. CD107a/LAMP1 expression on tumor-infiltrating CD8^+^ T cells was analyzed by flow cytometry after 12 days post-inoculation of B16-F10 cells. *N* = 8 (ctrl), 9 (cKO) Gating: All cells>singlets>live cells>CD8^+^ T cell>CD107a. **f** NK cell degranulation assay by measuring CD107a/LAMP1 expression on tumor-infiltrating NK cells. *N* = 8 (ctrl), 9 (cKO). Gating: All cells>singlets>live cells>NK cells>CD107a. **g** CD4^+^ T cell degranulation assay by measuring CD107a/LAMP1 expression on CD4^+^ T cells. *N* = 8. Gating: All cells>singlets>live cells>CD4^+^ T cell>CD107a. **h** Flow cytometric analysis to show the changes in B16-F10 subcutaneous tumor-infiltrating Treg (CD4^+^CD3e^+^FoxP3^+^) populations upon *Cyp11a1* deletion. *N* = 9. **i**–**l** Comparing immunophenotype in Cyp11a1 inhibitor, AG, treated and untreated mice with B16-F10 cells injected subcutaneously. All are representative of two independent experiments. **i** Representative FACS profile to show iNOS and Arg1 expression in tumor-infiltrating CD45^+^Lin^−^CD11b^+^F4/80^+^ macrophages (left panel). Graphical presentation the iNOS and Arg1 expression of a representative experiment (right panel). *N* = 5. **j**–**l** CD107a/LAMP1 expression on CD4^+^ T cells (**j**), CD8^+^ T cells (**k**) and NK cells (**l**) was analyzed by flow cytometry after 12 days post-inoculation of B16-F10 cells. *N* = 4 (ctrl), 3 (cKO). Gating: All cells>singlets>live cells>CD4^+^ or CD8^+^ T or NK cells>CD107a. In this figure, all error bars represent mean with s.d. *P*-value was calculated by unpaired two-tailed *t*-test. *N* represents biologically independent animals.

Steroid hormones can induce an immunosuppressive M2-like phenotype in macrophages^26,42,43^ cell death and anergy in T cells^26,44–46^, tolerance in dendritic cells^26,47–49^ and increase the frequency of Treg cells^50–52^. Therefore, we tested whether steroidogenic T cells support tumor growth through the induction of immunosuppressive phenotypes in infiltrating immune cells. To determine whether intratumoral macrophages were M1 or M2 type, we purified tumor-infiltrating macrophages and analyzed mRNA expression of the M2-macrophage signature genes *Arg1* and *Tgfβ1*. In tumor-infiltrating macrophages from *Cyp11a1* cKO mice, *Arg1* and *Tgfβ1* mRNA expression was significantly reduced compared to the control mice, indicating fewer of the tumor-supporting M2 macrophages in the T cell-specific *Cyp11a1* knockout mice (Fig. 6a). We confirmed changes in macrophage characteristics at the protein level, showing a significant decrease in Arg1^+^ macrophages (M2) with coincident increase in tumor-infiltrating iNOS^+^ macrophages (M1) upon T cell-specific *Cyp11a1* deletion (Fig. 6b, c). Hence, depletion of Cyp11a1 in T cells increases the M1/M2 macrophage ratio in the TME.

Consistent with enhanced tumor immunity, we found significantly higher levels of the inflammatory cytokines *Ifnγ* and *Tnfα* expression in CD8^+^ T cells following *Cyp11a1* ablation (Fig. 6d). Moreover the expression of the co-inhibitory receptors^53^ PD1 and TIGIT on tumor-infiltrating T cells was reduced in CD4^+^ TILs in the *Cyp11a1* cKO mice compared with control littermates (Supplementary Fig. 5a). The data indicate greater T cell functionality and less exhaustion in the *Cyp11a1* cKO mice.

To test cytotoxic capacity of T and NK cell populations, we examined the degranulation response of these cells by analyzing cell surface expression of CD107a/LAMP1 in tumor-infiltrating T and NK cells. We observed a significantly increased proportion of degranulating CD107a^+^CD8^+^ T cells in *Cyp11a1* cKO mice compared to control mice (Fig. 6e). The degranulation response in NK cells and CD4^+^ T cells was also enhanced (Fig. 6f, g).

Finally, we observed that the proportion of intratumoral Tregs was decreased (Fig. 6h). Altogether these data suggest that inhibition of T cell steroidogenesis by genetic deletion of *Cyp11a1* changes immune cell composition in the tumor microenvironment in favor of anti-tumor immunity.

Similar to the genetic deletion of *Cyp11a1*, application of the Cyp11a1 inhibitor aminoglutethimide significantly increased frequencies of M1 macrophages and degranulating T and NK cells (Fig. 6i–l, Supplementary Fig. 5b), recapitulating anti-tumor phenotypes. *Cyp11a1* inhibition also decreased the number of M2 macrophages in the tumor (Fig. 6i). Together, these data demonstrate the potential to stimulate anti-tumor immunity through pharmacological suppression of *Cyp11a1-*dependent steroidogenesis pathways.

## Discussion

**Fig. 7:**
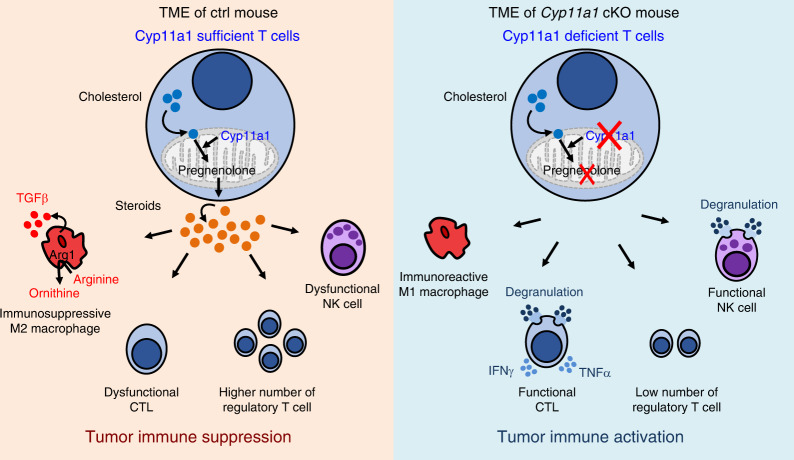
Graphical abstract of the discovery. T cell-mediated de novo steroidogenesis in the tumor microenvironment promotes tumor growth by inhibiting anti-tumor immunity, in part by inducing M2 phenotype in macrophages and suppressing T and NK cell function. Genetic deletion of *Cyp11a1* or pharmacologic inhibition of Cyp11a1 activity stimulates anti-tumor immunity by increasing the number of M1 macrophages and functional T cells, and decreasing the number of regulatory T cells, M2 macrophages.

The endocrine importance of systemic steroid hormones is well documented in regulating cell metabolism and immune cell function but the intracrine, autocrine, and paracrine role of local cell-type-specific steroidogenesis is less clear, particularly in pathologies such as cancer^1,2^. This is in part due to the lack of tools to study steroidogenesis in a tissue-specific manner in vivo. To overcome this, we generated a *Cyp11a1*-mCherry reporter and a conditional *Cyp11a1* knockout mouse strain to identify de novo steroidogenic cells and study their role in vivo. Using these discovery tools, complemented by pharmacological intervention, we uncovered an anti-tumor immune suppression mechanism that may be exploited clinically to boost the anti-tumor immunity (Fig. 7).

The TME harbors innate immune cells (such as macrophages, neutrophils, eosinophils, mast cells, myeloid derived suppressor cells, dendritic cells, and natural killer cells), adaptive immune cells (such as T and B cells), stromal cells (such as fibroblasts, endothelial cells, pericytes, mesenchymal cells), and cancer cells. The presence of immune cells in the tumor microenvironment raises a long-standing question: How tumors evade the immune surveillance and anti-tumor immunity? The present understanding suggests that the balance between protective anti-tumoral immune cells (and other protective factors) and pro-tumoral immunosuppressive cells (and other immunosuppressive factors) determines the fate. Anti-tumoral immune cell subsets includes Th1, Tc1 (CD8^+^ CTL), M1 macrophage, DC1 which are antagonized by Th2, M2, tolerogenic DC, immature DC, DC2, Treg, and myeloid-derived suppressor cells. Established tumors often foster immunosuppressive and tolerogenic immune cells. In this report, the discovery of immune cell-mediated steroid biosynthesis in the TME explains how an immunosuppressive TME can be achieved. Previously it has been shown that steroids induce anergy and cell death in T cells^44–46,54^, M2 phenotype in macrophages^42,43^, tolerance in DCs^47–49^, and increase the frequency of Treg cells^50–52^. Here we demonstrate that tumor-infiltrating T cells can produce steroids and that the inhibition of T cell-mediated steroid biosynthesis tips the balance toward protective immunity. We have shown that ablation of intratumoral steroids by deleting Cyp11a1 in T cells reduced the number of M2 macrophages and Tregs, and increased the number of M1 macrophages, functional T cells, and NK cells. The results suggest that the secreted steroid works in a paracrine manner that effect on other infiltrating immune cells as expected. All these cell types are known to be crucial in regulating anti-tumor immunity and immunosuppression. Therefore, in the absence of intratumoral steroids, anti-tumor immunity is more effective and restricts tumor progression. Importantly, this pathway can be targeted pharmacologically. This may be exploited therapeutically in three ways: (1) steroidogenic gene expression or the presence of steroids might be used to stratify the patients, (2) use of pharmacologic drug (i.e. anti-steroidogenic) can convert the TME into protective type-1 inflammatory condition by reducing the steroid level, and (3) immunotherapy should work better in absence of immunosuppressive steroids.

Single-cell level transcriptomic analysis revealed important findings pertaining to tumor immune infiltrates and provide a rich resource for cancer immunologists. We identified three distinct classes of immune cells with steroidogenic potential, T cells (Th2 and Tc2, major population), mast cells (minor population), basophils (minor population), and a few monocyte/macrophages. GATA factors are revealed as key drivers of Cyp11a1 expression in immune cells. In Th2 cells Gata3 occupies the *Cyp11a1* locus. Cyp11a1^+^ Th2 cells outnumbered other types of Cyp11a1-expressing cells. Hence deletion of *Cyp11a1* in T cells was sufficient to remodel the TME for more effective anti-tumor immune responses. In the in vitro setting, we observed that Tregs and other T helper cell types (except Th1) can upregulate Cyp11a1 expression, but in the in vivo setting we noticed that only Th2 cells were Cyp11a1^+^. We did not find Cyp11a1-expressing Tregs or any other T helper cell types in the B16-F10 TME. The presence of Cyp11a1^+^ non-T cells indicates that in a different context, such as in different tumor types, the steroidogenic population may vary and other hematopoietic cells may also produce steroids. Future studies are essential to  gain detailed insight of immune cell-mediated intratumoral steroidogenesis in different tumor type. It would be interesting to know why CD4^+^ T cells upregulate Cyp11a1 expression specifically in the TME, and not in the secondary lymphoid organs. This could be a result of the fact that Th2 cells are numerous in tumors but not in the spleen and lymph nodes. Therefore, further studies are needed to delineate whether this is a result of an increased Th2 cell abundance in tumors, and/or an enhanced Cyp11a1 expression per individual Th2 cell.

The induction of type-2 immune response in tumors such as breast, pancreatic, melanoma, glioma, cervical, lung, and bladder cancer has been previously reported, both in humans and mice, and its roles in the promotion of cancer by immunosuppression are known^12–25^. As expected, reanalysis of publicly available transcriptomic datasets showed evidence of many human tumor types expressing steroidogenic genes. In melanoma, we show evidence of steroidogenic gene expression being correlated with type-2 immune signature genes such as *IL4*. The limitation of this analysis is that it does not confirm the cellular source of *CYP11A1* expression because the transcriptomes were obtained from bulk RNAseq of whole tumors. In the future, single-cell RNA sequencing of tumor-infiltrating Th2 cells from patients may provide direct evidence and further insights. Analysis of publicly available single-cell RNA sequencing datasets of human tumor-infiltrating T cells revealed that all the available datasets are from tumors that predominantly raised Th1-mediated immune responses. Therefore, as expected, we observed no or only a few *CYP11A1*-expressing cells in these data sets (data not shown). This is consistent with our hypothesis in the sense that we expect *CYP11A1* expression predominantly in tumors exhibiting type-2 immune responses.

The study adds to the concern raised by recent studies about proper use of steroids, particularly glucocorticoids, in solid tumors as a part of the disease management, specifically when we seek to boost anti-tumor immune response^55–62^. Glucocorticoids have been used in clinical oncology for over a half a century, and at present these are routinely used to alleviate edema in patients with intracranial lesions and are first-line agents to suppress immune-related adverse events that arise with the immunotherapies. Therefore, further studies are required to evaluate the use of synthetic steroids if the tumors itself induces glucocorticoid synthesis. Reduction of intratumoral steroidogenesis may open a window for immunotherapies to work better at lower doses.

Similar experimental approaches can in future provide in depth mechanistic insights into other extraglandular (local) steroidogenic cell types such as adipose cells, neuron, osteoblasts, astrocytes, microglia, skin, trophoblast, and thymic epithelial cells. Our *Cyp11a1*-mCherry reporter mouse line can be used as a discovery tool to identify undiscovered steroidogenic cell types in tolerogenic physiological conditions such as pregnancy, mucosal tolerance, and inflammatory and immunopathological conditions. Their functional role can be dissected by using *Cyp11a1* cKO mice using tissue-specific Cre-drivers. Nevertheless, cancer is a proven context where tumors hijack this pathway for immune evasion. Further studies in diverse physiological scenarios would be of great interest to more broadly understand the role of immune cell de novo steroidogenesis in regulating inflammation and immunity.

## Methods

### Mice

The use of all mice in this research article were in accordance with the UK Animals in Science Regulation Unit’s Code of Practice for the Housing and Care of Animals Bred, Supplied or Used for Scientific Purposes, the Animals (Scientific Procedures) Act 1986 Amendment Regulations 2012. All experimental procedures were performed under UK Home Office Project licenses (PPL 80/2574 or PPL P8837835 or PPL P6B8058B0), which were reviewed and approved by the Medical Research Council Laboratory of Molecular Biology (PPL 80/2574 or PPL P8837835) and Wellcome Sanger Institute (PPLP6B8058B0) Animal Welfare and Ethical Review Bodies (AWERB). Sample sizes were determined based on previous experience and a priori power analysis (G* Power). Housing conditions at the Sanger Institute: all the mice used in this study, were maintained in specific pathogen-free unit on a 12-h light and 12-h dark cycle (lights off at 7:30 pm and no twilight period). The ambient temperature was 21 °C with a maximum variation of ±2 °C. The humidity was 55 ± 10%. Mice were housed for phenotyping with a stocking density of 3–5 mice/cage (dimensions of caging: (L × W × H) 365 × 207 × 140 mm, floor area 530 cm^2^) in individually ventilated caging (Tecniplast Seal Safe1284L) receiving 60 air changes/h. In addition to Aspen bedding substrate, we provide standard environmental enrichment of two nestlets, a cardboard Fun Tunnel and three wooden chew blocks. Given water and diet were available all the times. Mice were fed on Mouse Breeders Diet (Lab Diets, 5021-3). Animals recruited to studies at ARES Medical Research Council animal facility remained socially housed in individually ventilated cages, at ambient temperature and with cage enrichment. Animals of each genotype were randomly assigned to experimental groups, and where possible, technicians performing the experiment were blinded to experimental groups and treatments.

### Generation of a Cyp11a1-mCherry reporter mouse line

Using CRISPR_Cas9 technology we introduced double-strand DNA breaks 5′ and 3′ adjacent to the *Cyp11a1* termination codon in exon 9 to facilitate the introduction of our targeting construct. The 5′ and 3′ arms of homology were designed to remove the *Cyp11a1* termination codon and 100 bp of the 3′ UTR immediately downstream and replace it with a minimal T2a self-cleavage peptide followed by the fluorescent marker mCherry. Using the web-based tool designed by Hodgkins et al.^63^, two sgRNAs were identified 5′ and 3′ adjacent to the *Cyp11a1* termination codon. The guide sequences were ordered from Sigma Genosys as sense and antisense oligonucleotides, and annealed before individually cloning into a human U6 (hU6) expression plasmid (Addgene # 41824). Next, we generated targeted ES cell (ESCs) through nucleofection of 3 × 10^6^ JM8 ESCs with 2 μg *Cyp11a1* circular targeting construct, 1.5 μg of each hU6_sgRNAs, and 3 μg a plasmid expressing human codon-optimized CAS9 driven by with the CMV promoter (Addgene # 41815). 48 h post transfection the media was changed for G418 selection media (165 ug/ul). Confirmation of the correct targeting events were confirmed by quantitative PCR (qPCR) for loss of heterozygosity (LOH) and the presence of the selectable marker, and long range (LR) PCR and Sanger sequencing. Following blastocyst injection and chimaera breeding three F1 *Cyp11a1* ^+/^^*mCherry-Neo*^ mice were bred to *pCAGGSs-Flpo*^64^ mice to remove the neomycin selectable marker to generate *Cyp11a1*^*+/mCherry*^ mice.

Genotyping primer sequence (Supplementary Fig. 1a)

GF ATGGTGACACAGACCTCCTTG

GR CCCAGGAACAGAAAGGCTTG

R2R AGGCGCATAACGATACCACG

R1R GGATTCTCCTCCACGTCACCG

### Generation of a Cyp11a1 cKO mice

*Cyp11a1*^*fl/fl*^ mice were generated by crossing *Cyp11a1*^*tm1a(KOMP)Wtsi*^ mice with a previously reported Flp-deleter (FlpO) line^64^. *Cyp11a1*^*fl/fl*^ mice were crossed with *Cd4-cre* mice to generate the *Cyp11a1* cKO mice.

### Syngeneic mouse tumor models

B16-F10 melanoma model: the C57BL/6 derived B16-F10 melanoma cell line was purchased from American Type Culture Collection (ATCC) and cultured in Dulbecco’s Modified Eagle medium (DMEM, Life Technologies), supplemented with  100 U/mL penicillin/streptomycin and 10%  fetal bovine serum (FBS; Life Technologies, Invitrogen). For the primary tumor growth assay, 2.5 × 10^5^ B16-F10 cells were injected subcutaneously into the shoulders of either wild type (WT) C57BL/6 mice, *Cd4-Cre, Cyp11a1*^*fl/fl*^ or *Cd4-Cre;Cyp11a1*^*fl/fl*^ mice. Once tumors were palpable, non-invasive tumor measurements were recorded daily and volumes calculated using the following formula (*π*/6) (shortest length × longest length). After 11 or 12 days animals were killed and tissues collected for analysis. For the experimental metastasis assay, 5 × 10^5^ B16-F10 cells in a volume of 0.1 ml PBS were injected intravenously into the tail vein. After 10 days (±1 day) the mice were killed via cervical dislocation, and their lungs removed and rinsed in phosphate-buffered saline. The number of B16-F10 colonies on all five lobes of the lung were counted macroscopically. Exogenous pregnenolone treatment: On days 5, 7, and 9, tumor-bearing mice received 10 μl of vehicle or pregnenolone (20 mg/ml) in DMSO via topical application at tumor site. Tumor volumes were recorded until the experimental endpoint on day 12. Orthotopic EO771 breast cancer model: the EO771 breast cancer cell line was purchased from CH3 BioSystems and cultured in RPMI (Sigma) supplemented with 10% FBS, 1% PS, and 10 mM HEPES. In all, 2.5 × 10^5^ cells were injected into the 4th inguinal mammary fat pad of *Cyp11a1*-mCherry reporter mice. Tumors and inguinal lymph node were collected after 15 days of tumor development.

Lung colonization of EO771 cells: 4 × 10^5^ cells were injected intravenously in to the tail vein. After 10 days (±1 day) the mice were killed via cervical dislocation, and their lungs were removed, rinsed in PBS and fixed in 10% neutral buffered formalin. The fixed lungs were paraffin embedded and sections stained with Hematoxylin and Eosin using routine histology protocols. The microscopic images were captured in Olympus with ×10 magnification and the number of metastatic foci were counted. Treatment with aminoglutethimide (AG): for both the B16-F10 and E0771 models 200 μl of a 2-mg/ml AG or vehicle were administered via the intraperitoneal route. The first dose was administered concurrently with cell implantation and repeated every 2 days thereafter.

### Tumor tissue processing

Tumors were mechanically dissociated and digested in 1 mg/ml collagenase D (Roche), 1 mg/ml collagenase A (Roche), and 0.4 mg/ml DNase I (Sigma) in IMDM media containing 10% FBS, at 37 °C for 40 mins. EDTA was added to all samples to neutralize collagenase activity (final concentration 5 mM) and digested tissues were passed through 70μm filters (Falcon).

### Cell sorting

Once processed, single-cell suspension tumor samples were incubated with a fixable fluorescent viability stain (Life Technologies) for 20 mins (diluted 1:1000 in PBS) prior to incubation with conjugated primary antibodies for 30 mins at 4 °C. Antibodies were diluted in PBS with 0.5% bovine serum albumin (BSA). Stained samples were sorted, using the MoFlo XDP or BD Influx cytometer system.

### T helper cell culture

Splenic naïve T helper cells from *Cyp11a1*-mCherry reporter mice were purified with the CD4^+^CD62L^+^T Cell Isolation Kit II (Miltenyi Biotec) and polarized in vitro toward differentiated Th1, Th2, Th9, Th17, iTreg, and Tfh subtype following Pramanik et al.^65^. Naïve cells were seeded into anti-CD3e (2 μg/ml, clone 145-2C11, eBioscience) and anti-CD28 (5 μg/ml, clone 37.51, eBioscience) coated plates. The medium contained the following cytokines and/or antibodies as described below. To culture *Th1 subtype*: recombinant murine IL2 (10 ng/ml, R&D Systems), recombinant murine IL12 (10 ng/ml, R&D Systems), and neutralizing anti-IL4 (10 μg/ml, clone 11B11, eBioscience). *Th2 subtype:* Recombinant murine IL2 (10 ng/ml, R&D Systems), recombinant murine IL-4 (10 ng/ml, R&D Systems), and neutralizing anti-IFNg (10 μg/ml, clone XMG1.2, eBioscience). *Th9 subtype:* 20 ng/ml recombinant mouse IL4, 2 ng/ml recombinant human TGFb, and 10μg/ml neutralizing anti-IFNγ. *Th17 subtype:* 30 ng/ml recombinant mouse IL6, 5 ng/ml recombinant human TGFb, and 50 ng/ml recombinant mouse IL23. *Tfh subtype:* 50 ng/ml recombinant mouse IL21, 10 μg/ml neutralizing anti-IL4 and anti-IFNγ. *iTreg subtype*: 5 ng/ml recombinant mouse IL2, 5 ng/ml recombinant human TGFb. The cells were removed from the activation plate on day 4 (after 72 h). Th2 cells were cultured for another 2 days in the absence of CD3e and CD28 stimulation. Then, cells were restimulated by seeding on coated plate for 6  h. For flow cytometric detection cells were treated with monensin (2 μM, eBioscience) for the last 3 h.

### In vitro Tc1 and Tc2 differentiation

Splenic naïve CD8^+^ T cells were purified by using Naive CD8a^+^ T Cell Isolation Kit, mouse (Miltenyi Biotec) following the manufacturers protocol, and polarized in vitro toward differentiated Tc1 and Tc2. In brief, naive cells were seeded into anti-CD3e (2 μg/ml, clone 145-2C11, eBioscience) and anti-CD28 (5 μg/ml, clone 37.51, eBioscience) coated plates. The medium contained the following cytokines and/or antibodies:

*Tc1 subtype*: recombinant murine IL2 (10 ng/ml, R&D Systems), recombinant murine IL12 (10 ng/ml, R&D Systems) and neutralizing anti-IL4 (10 μg/ml, clone 11B11, eBioscience). *Tc2 subtype:* Recombinant murine IL2 (10 ng/ml, R&D Systems), recombinant murine IL-4 (10 ng/ml, R&D Systems) and neutralizing anti-IFNg (10 μg/ml, clone XMG1.2, eBioscience).

### Single-cell RNA sequencing

Single cells were isolated from processed B16-F10 subcutaneous tumors using a fluorescence-activated cell sorter. Once processed, tumor tissues were incubated with a fixable fluorescent viability stain (Live-Dead Violet, Life Technologies) for 15 mins (diluted 1:1000 in PBS) prior to incubation with conjugated primary antibodies for 30 mins at 4 °C in the dark. Antibodies were diluted in flow cytometry staining buffer (eBioscience/Thermo Fisher Scientific). After staining, samples were sorted using the BD influx flow cytometer. Single-cells were harvested in 2 μl of Lysis Buffer (1:20 solution with RNase Inhibitor (Clontech, cat. no. 2313 A) in 0.2% v/v Triton X-100 (Sigma-Aldrich, cat. no. T9284)) in 96-well plates. After sorting the plates were spun down quickly and kept frozen at −80 °C. Then we performed reverse transcription (RT) and cDNA pre-amplification following SmartSeq2 protocol to obtain mRNA libraries from single-cells. Oligo-dT primer, dNTPs (ThermoFisher, cat. no. 10319879) were then added to the samples. RT-PCR were performed using 50U of SMARTScribe™ Reverse Transcriptase (Clontech, cat. no. 639538). cDNA libraries were prepared using the Nextera XT DNA Sample Preparation Kit (Illumina, cat. no. FC-131-1096), following the protocol supplied by Fluidigm (PN 100^−^5950 B1). Single-cell libraries were pooled, purified using AMPure XP beads (Beckman Coulter) and sequenced on an Illumina HiSeq 2500 aiming for an average depth of 1 million reads/cell (paired-end 100-bp reads).

### Single-cell RNA sequencing analysis

The sequencing data was mapped using STAR 2.5.1b and quantified using htseq 0.9.0. The data was subsequently processed in Scanpy 1.3.6 with the standard Seurat-inspired workflow. Cells with above 20% mitochondrial reads were removed, and the data was transformed to log(CPM/100 + 1) form. Highly variable genes were identified via normalized dispersion, with the top 10% of genes reported as variable. The data was filtered to highly variable genes, scaled and used for PCA. The top 20 principal components were used to infer a neighbor graph, which served as the basis of UMAP dimensionality reduction and Leiden clustering with a 0.4 resolution value. Per-cluster markers were identified using default Scanpy settings, using log(CPM/100 + 1) data on input. Genes correlated with *Cyp11a1* were identified with the Pearson Correlation Coefficient. Potential *Cyp11a1* regulators were detected via pySCENIC, replicating the demonstration notebook.

### ATAC-seq

Naïve T cells isolation and in vitro T helper cell differentiation were carried out as described above. After 72 h of continuous activation/differentiation, Th1, Th2, and Th17 cells were harvested. The ATAC-seq experiments were performed following the published protocol with some modification^66^. Briefly, 50,000 cells were washed with ice-cold 1X Dulbecco's PBS (twice), and resuspended in a sucrose swelling buffer (0.32 M sucrose, 10 mM Tris.Cl, pH 7.5, 3 mM CaCl_2_, 2 mM MgCl_2_, 10% glycerol). The cell suspension was incubated for 10 m on ice. In next, a final concentration of 0.5% NP-40 was added, and the cells suspension was vortexed for 10 s and incubated for 10 m on ice. Nuclei were pelleted at 500 × *g* at 4 °C for 10 m. Then, the nuclei pellets were washed once with 1X TD buffer (from Nextera DNA Library Preparation Kit, Illumina, #FC-121-1030), and resuspended in 50 ul tagmentation mixture that contains 25 ul 2X TD buffer (Nextera DNA Library Preparation Kit, Illumina #FC-121-1030), 22.5 ul H_2_O, 2.5 ul TDE1 (Nextera DNA Library Preparation Kit, Illumina #FC-121-1030). The tagmentation reaction was performed on an Eppendorf ThermoMixer C at 37 °C, 800 rpm, for 30 m. The reaction was stopped by adding 250 μl (5 vol) Buffer PB (QIAGEN MinElute PCR Purification Kit). The tagmented DNA was purified by QIAGEN PCR Purification Kit according to manufacturer’s instructions and eluted in 12.5 μl Buffer EB from the kit, which yielded ~10 μl purified DNA. The library amplification was performed in a 25-μl reaction that includes 10 μl purified DNA (from above), 2.5 μl PCR Primer Cocktail (Nextera DNA Library Preparation Kit, Illumina #FC-121-1030), 2.5 μl N5xx (Nextera index kit, Illumina #FC-121-1012), 2.5 μl N7xx (Nextera index kit, Illumina #FC-121-1012), and 7.5 μl NPM PCR master mix (Nextera DNA Library Preparation Kit, Illumina #FC-121-1030). PCR reaction condition was: 72 °C 5 m, 98 °C 2 m, [98 °C 10 s, 63 °C 30 s, 72 °C 60 s] × 12, 10 °C hold. Amplified libraries were purified by double Agencourt AMPureXP beads purifications (Beckman Coulter, #A63882). 0.4X beads:DNA ratio for the first time, flow through was kept (removing large fragments); 1.4X beads: DNA ratio for the second time, beads were kept. Libraries were eluted from the beads in 20 μl Buffer EB (from QIAGEN PCR Purification Kit). In all, 1 μl library was run on an Agilent Bioanalyzer to check size distribution and quality of the libraries. Sequencing was done by an Illumina Hiseq 2500 (75 bp PE).

### ATAC-seq data processing

ATAC-seq reads were aligned using Bowtie 2^67^ with the parameter –X 2000 and the mouse genome mm10. This was followed by peak calling on each replicate individually using MACS2^68^ with the function callpeak and the parameters -B -SPMR -shift -100 -extsize 200. The peaks obtained were kept if they overlapped a peak from the other replicate of the same T helper cell types. The resulting bedGraph files generated by MACS2 were converted to bigWig format for the visualization on UCSC genome browser^69^.

### ChIP-seq data processing

GATA3 ChIP-seq data sets from different Tn subtypes were downloaded from the NCBI GEO database (GSE20898)^70^. The SRA files were converted to fastq files using the SRA toolkits (http://ncbi.github.io/sra-tools/). Reads were aligned to the mm10 genome using Bowtie 2^67^ with default parameters. Peak calling was performed by using MACS2^68^ with the function callpeak and the parameters -B -SPMR -nomodel -extsize 200. The resulting bedGraph files generated by MACS2 were converted to bigWig format for the visualization on UCSC genome browser^69^.

### B16-F10 and T cell co-culture assay

Negatively purified splenic naïve CD4^+^ T cells from *Cyp11a1*-mCherry mice were cultured in presence of B16-F10 cell with or without TCR activation and analyzed by flow cytometry to detect Cyp11a1-mCherry expression. Naïve T cell purification and activation condition is described in the T helper cell culture section above. Briefly, naïve splenic cells were activated in anti-CD3e and anti-CD28 coated plate in presence of IL2 but absence anti-IL4 neutralizing antibody.

### Quantitative PCR

Tumor-infiltrating macrophages (Lin^−^CD11b^+^) and CD8^+^ T cells were purified by cell sorting. We used the Cells-to-C_T_ kit (Invitrogen/Thermofisher Scientific) and followed SYBR Green format according to manufacturer’s instructions. In all, 2 μl of cDNA was used in 12 μl qPCR reactions with appropriate primers and SYBR Green PCR Master Mix (Applied Biosystems). Data were analyzed by ddCT method. Experiments were performed three times and data represent mean values ± standard deviation. The primer list is provided below:

*Arg1*: F- ATGGAAGAGACCTTCAGCTAC

R- GCTGTCTTCCCAAGAGTTGGG

*Tgfβ1*: F- TGACGTCACTGGAGTTGTACGG

R- GGTTCATGTCATGGATGGTGC

*Ifnγ*: F- ACAATGAACGCTACACACTGC

R- CTTCCACATCTATGCCACTTGAG;

*Tnfα*: F- CATCTTCTCAAAATTCGAGTGACAA

R- TGGGAGTAGACAAGGTACAACCC

*Gapdh*: F- ACCACAGTCCATGCCATCAC

R- GCCTGCTTCACCACCTTC

*Rplp0*: F: CACTGGTCTAGGACCCGAGAA

R: GGTGCCTCTGGAGATTTTCG

### Flow cytometry

We followed eBioscience surface staining, intracellular cytotoplasmic protein staining (for cytokines) protocols. Briefly, single-cell suspensions were stained with Live/Dead Fixable Dead cell stain kit (Molecular Probes/ Thermo Fisher) and blocked by purified rat anti-mouse CD16/CD32 purchased from BD Bioscience and eBioscience. Surface staining was performed in flow cytometry staining buffer (eBioscience) or in PBS containing 3% FCS at 4 °C. For intracellular cytokine staining cells were fixed by eBioscience IC Fixation buffer and permeabilized by eBioscience permeabilization buffer. Cells were stained in 1x permeabilization buffer with fluorescent dye-conjugated antibodies. After staining cells were washed with flow cytometry staining buffer (eBioscience) or 3% PBS-FCS, and were analyzed by flow cytometer Fortessa (BD Biosciences) using FACSDiva. The data were analyzed by FlowJo v10.2 software. Antibodies used in flow cytometry were: CD4 (RM4-5 or GK1.5), eBioscience, BD Bioscience and Biolegend. 1:400; CD8a (53-6.7), eBioscience 1:400; CD3e (145 2c11), eBioscience 1:400; TCRb (H57-597, BB790) BD Bioscience 1:400; CD45 (30F11), BD Bioscience 1:1000; CD44 (IM7), eBioscience 1:400; CD25 (PC61), BD Bioscience 1:400; B220 (Ra3-6b2), eBioscience 1:400; Ly6G (1A8), eBioscience 1:400; Ly6G/Ly6C/Gr-1 (RB6-8C5), eBioscience 1:400; Ly6C (HK1.4), eBioscience 1:400; SiglecF (E50-2440) BD Bioscience 1:1000; CD11b (M1/70), eBioscience 1:400; CD11c (N418), eBioscience 1:400; CD19 (1D3), eBioscience 1:400; NK1.1 (Pk136), Biolegend 1:400; Ter119 (TER119), eBioscience 1:400; PD-1 (J43), eBioscience 1:400; TIGIT (1G9), BD Bioscience 1:400; CD107a/LAMP1 (1D4B), eBioscience 0.5 μg/test(100ul); IFNg (XMG1.2) PerCP-Cy5.5 eBioscience 1:1500 Cat no. 45-7311-82; IL4 (11B11) APC eBioscience 1:200 Cat no 17-7041-82; IL13 (eBio13a) AF488 eBioscience, 1:400, Cat. no. 53-7133-82; IL17 (eBio64DEC17), APC, 1:200, Cat. no. 17-7179-42; CD117(2B8) BV711, BD Bioscience, cat no. 563160.

### Western blot antibodies

Anti-CYP11A1 (Santa Cruz Biotechnology, C-16) and anti-TBP (Abcam) were used.

### Quantitative ELISA

CD45^+^ leukocytes were purified from B16-F10 tumor masses and lungs, of mice that had been tail vein administered B16-F0 cells, and seeded at equal density in IMDM medium supplemented with 10% charcoal stripped FBS (Life Technologies, Invitrogen) for 24 h. Pregnenolone concentrations of the culture supernatants were quantified using pregnenolone ELISA kit (Abnova) and corticosteroids ELISA (Thermofisher) kit following the manufacturers’ instruction. Absorbance was measured at 450 nm, and data were analyzed in GraphPad Prism 6.

### T cell proliferation assay

Negatively purified splenic naïve CD4^+^ T cells were purified from *Cyp11a1* cKO, *Cd4*-Cre and *Cyp11a1*^*fl/fl*^ mice, stained with CellTrace Violet following the CellTrace Violet Cell Proliferation Kit (Invitrogen) protocol, activated in vitro, and the cell proliferation profile was captured by flow cytometry based dye decay assay on BD Fortessa^65^. Data were analyzed in FlowJo v9.

### Reporting summary

Further information on research design is available in the Nature Research Reporting Summary linked to this article.

## Supplementary information

Supplementary Information

Peer Review File

Description of Additional Supplementary Files

Supplementary Data 1

Supplementary Data 2

Reporting Summary

## Data Availability

scRNAseq data is deposited to ArrayExpress with accession number E-MTAB-8509 [https://www.ebi.ac.uk/arrayexpress/experiments/E-MTAB-8509/]. The ATACseq data (exclusively generated for this study) is in ENA and deposited within a large study name with many other data sets. The accession number in ENA is PRJEB14081 [https://www.ebi.ac.uk/ena/data/search?query=PRJEB14081]. The sample info and run numbers referring to the ATAC-seq data we used in the manuscript can be found in the spreadsheet provided as Supplementary Data 1. GATA3 ChIP-seq data sets from different Tn subtypes were downloaded from the NCBI GEO database (GSE20898) [https://www.ncbi.nlm.nih.gov/geo/query/acc.cgi?acc=GSE20898]. The raw data source of Fig. 3j was GSE19234^71^ [https://www.ncbi.nlm.nih.gov/geo/query/acc.cgi?acc=GSE19234] and Fig. 2l was EGAD00001000325^72^ [https://www.ebi.ac.uk/ega/datasets/EGAD00001000325]. Access to EGAD00001000325 dataset is available upon request to the Data Access Committee at datasharing@sanger.ac.uk. In Supplementary Fig. 2g we searched “Expression Atlas (EMBL-EBI)” with search query gene name “Cyp11a1”, species “Homo sapiens”, “cancer” as disease condition, baseline expression, arranged by expression rank, downloaded data, rebuilt the figure, excluded ovarian cancer to avoid confusion. Link for the search is provided below: https://www.ebi.ac.uk/gxa/search?geneQuery=%5B%7B%22value%22%3A%22Cyp11a1%22%7D%5D&species=homo%20sapiens&conditionQuery=%5B%7B%22value%22%3A%22cancer%22%7D%5D&bs=%7B%22homo%20sapiens%22%3A%5B%22DISEASE%22%5D%7D&ds=%7B%22kingdom%22%3A%5B%22animals%22%5D%7D#baseline. Supporting data for the Supplementary Fig. 4j–i can be found in the Supplementary Data 2. All remaining relevant data are available in the article, supplementary information, or from the corresponding author upon reasonable request.
