# Supplementary material for: Tumors induce de novo steroid biosynthesis in T cells to evade immunity
Source: Nat Commun. 2020 Jul 17;11:3588. doi: 10.1038/s41467-020-17339-6 (PMC7368057; doi:10.1038/s41467-020-17339-6)
Supplement: Supplementary file 3 — Description of Additional Supplementary Files [file 41467_2020_17339_MOESM3_ESM.pdf]

Description of Additional Supplementary Files

Supplementary Data 1

Description: ATACseq Datasets that were used to generate Figure 4f. The sample information and run numbers referring to the ATACseq data we used in the manuscript (Figure 4f) are provided in this spreadsheet.

Supplementary Data 2

Description: Pairwise comparison of the clusters (supporting data of the Supplementary Figure 4i and j)
