# Supplementary material for: Tumors induce de novo steroid biosynthesis in T cells to evade immunity
Source: Nat Commun. 2020 Jul 17;11:3588. doi: 10.1038/s41467-020-17339-6 (PMC7368057; doi:10.1038/s41467-020-17339-6)
Supplement: Supplementary file 6 — Reporting Summary [file 41467_2020_17339_MOESM6_ESM.pdf]

nature research

Corresponding author(s): Sarah A. Teichmann

Last updated by author(s): 28th May 2020

Reporting Summary

Nature Research wishes to improve the reproducibility of the work that we publish. This form provides structure for consistency and transparency in reporting. For further information on Nature Research policies, see Authors & Referees and the Editorial Policy Checklist .

Statistics

For all statistical analyses, confirm that the following items are present in the figure legend, table legend, main text, or Methods section.

n/a	Confirmed	
☐	☒	The exact sample size (*n*) for each experimental group/condition, given as a discrete number and unit of measurement
☐	☒	A statement on whether measurements were taken from distinct samples or whether the same sample was measured repeatedly
☐	☒	The statistical test(s) used AND whether they are one- or two-sided<br>*Only common tests should be described solely by name; describe more complex techniques in the Methods section.*
☒	☐	A description of all covariates tested
☐	☒	A description of any assumptions or corrections, such as tests of normality and adjustment for multiple comparisons
☐	☒	A full description of the statistical parameters including central tendency (e.g. means) or other basic estimates (e.g. regression coefficient) AND variation (e.g. standard deviation) or associated estimates of uncertainty (e.g. confidence intervals)
☐	☒	For null hypothesis testing, the test statistic (e.g. *F*, *t*, *r*) with confidence intervals, effect sizes, degrees of freedom and *P* value noted<br>*Give P values as exact values whenever suitable.*
☒	☐	For Bayesian analysis, information on the choice of priors and Markov chain Monte Carlo settings
☒	☐	For hierarchical and complex designs, identification of the appropriate level for tests and full reporting of outcomes
☒	☐	Estimates of effect sizes (e.g. Cohen's *d*, Pearson's *r*), indicating how they were calculated

Our web collection on statistics for biologists contains articles on many of the points above.

Software and code

Policy information about availability of computer code

Data collection	FACS DIVA in the flow analyser BD Fortessa. Cell sorter XDP, BD Influx, Illumina HiSeq 2500,
Data analysis	FlowJo software v10.2 (TreeStar) except Figure 2d where FlowJo v9 used. Graph Pad Prism 6. Bowtie 2, MACS2, STAR 2.5.1b , Scanpy 1.3.6 with the standard Seurat-inspired workflow, pySCENIC, Genevestigator.

For manuscripts utilizing custom algorithms or software that are central to the research but not yet described in published literature, software must be made available to editors/reviewers. We strongly encourage code deposition in a community repository (e.g. GitHub). See the Nature Research guidelines for submitting code & software for further information.

Data

Policy information about availability of data

All manuscripts must include a data availability statement. This statement should provide the following information, where applicable:

- Accession codes, unique identifiers, or web links for publicly available datasets
- A list of figures that have associated raw data
- A description of any restrictions on data availability

scRNAseq data is deposited to ArrayExpress. Accession number E-MTAB-8509. Link: https://www.ebi.ac.uk/arrayexpress/experiments/E-MTAB-8509/ . The ATACseq data (exclusively generated for this study) is in ENA and deposited within a large study name with many other data sets. The accession number in ENA is PRJEB14081. Link: https://www.ebi.ac.uk/ena/browser/view/PRJEB14081 . The sample info and run numbers referring to the ATAC-seq data we used in the manuscript can be found in the spreadsheet (ATACseq_Th_subtypes) provided as additional information. The raw data source of Figure 3j was GSE19234, and Figure 2l was EGAD00001000325. In Supplementary Figure 2g we searched "Expression Atlas (EMBL-EBI)" with search query gene name "Cyp11a1", species "Homo sapiens", "cancer" as disease condition, baseline expression, arranged by expression rank, downloaded data, rebuilt the figure, excluded ovarian cancer to avoid confusion. Link for the search is provided below: https://www.ebi.ac.uk/gxa/search?geneQuery=%5B%7B%22value%22%3A%22Cyp11a1%22%7D%5D&species=homo%20sapiens&conditionQuery=%5B%7B%22value%22%3A%22cancer%22%7D%5D&bs=%7B%22homo%20sapiens%22%3A%5B%22DISEASE%22%5D%7D&ds=%7B%22kingdom%22%3A%5B%22animals%22%5D%7D#baseline

Field-specific reporting

Please select the one below that is the best fit for your research. If you are not sure, read the appropriate sections before making your selection.

☒ Life sciences ☐ Behavioural & social sciences ☐ Ecological, evolutionary & environmental sciences

For a reference copy of the document with all sections, see nature.com/documents/nr-reporting-summary-flat.pdf

Life sciences study design

All studies must disclose on these points even when the disclosure is negative.

Sample size	Where relevant sample size were determined using power calculations based on variability observed in prior experiments of a similar kind. In some experiment prior experience of sample size requirement was used to design experimental group sizes. For experiments where technical limitations prevented adequate statistical power to be obtained from single experiment from multiple experiments were pooled to provide sufficient statistical power.
Data exclusions	Pre-established exclusion criteria across samples from a given experiment were used to avoid subjective bias. Flow-cytometry samples which had undergone technical failure during processing or which had insufficient numbers of cells within relevant "all cells" (the first FSC/SSC gate) gate were excluded from analysis. In Supplementary Figure 3g, we excluded tumors of steroidogenic tissues such as tumours of ovary, testis and adrenal gland. We excluded these steroiodgenic tissues to avoid confusion. These tissues are known to synthesize steroids de novo. This exclusion criteria was pre-established.
Replication	Experiments were repeated and detailed in the Figure legends.
Randomization	Genetically altered mice were allocated based on genotype, GA compared with control litter mates.. Mice were age and sex-matched whenever possible.
Blinding	Researchers and animal technicians were blinded for the duration of experiments where possible. Animal IDs were then linked back to genotype at the conclusion of each experiment.

Reporting for specific materials, systems and methods

We require information from authors about some types of materials, experimental systems and methods used in many studies. Here, indicate whether each material, system or method listed is relevant to your study. If you are not sure if a list item applies to your research, read the appropriate section before selecting a response.

Materials & experimental systems

n/a	Involved in the study	
☐	☒	Antibodies
☐	☒	Eukaryotic cell lines
☒	☐	Palaeontology
☐	☒	Animals and other organisms
☒	☐	Human research participants
☒	☐	Clinical data

Methods

n/a	Involved in the study	
☒	☐	ChIP-seq
☐	☒	Flow cytometry
☒	☐	MRI-based neuroimaging

Antibodies

Antibodies used

Neutralizing antibodies
IFNg (clone XMG1.2, eBioscience, Cat. no. 16-7311-85)
IL4 (Clone 11B11, eBioscience, Cat. no. 16-7041-85)
IL12 (Clone C8.6, eBioscience, Cat. no. 16-7129-85)

Unconjugated
CD3e (clone 145-2C11, eBioscience, Cat. no. 16-0031-86)
CD28 (clone 37.51, eBioscience, Cat. no.16-0281-86)
Cyp11a1 (C-16, Santa Cruz, sc-18043)
Tata binding protein TBP (mAbcam 51841, ab51841, Abcam)
anti-mouse CD16/CD32 (2.4G2), BD Pharmingen, Cat. no. 553141, eBioscience Cat no 14-0161-82
HRP conjugated anti-goat secondary used in Cyp11a1 western blot (Biorad, Cat no.1721034, Abcam, Cat no. ab97110)

Following anti-mouse fluorescent dye conjugated antibodies were purchased from eBioscience, BD Bioscience or Biolegend

(Clones are written within parentheses).
CD4 (RM4-5 or GK1.5), eBioscience, BD Bioscience and Bio legends. 1:400
CD8a (53-6.7), eBioscience 1:400
CD3e (145-2c11), eBioscience 1:400
TCRb (H57-597, BB790) BD Bioscience 1:400
CD45 (30F11), BD Bioscience 1:1000
CD44 (IM7), eBioscience 1:400
CD25 (PC61), BD Bioscience 1:400
B220 (Ra3-6b2), eBioscience 1:400
Ly6G (1A8), eBioscience 1:400
Ly6G/Ly6C/Gr-1 (RB6-8C5), eBioscience 1:400
Ly6C (HK1.4), eBioscience 1:400
SiglecF (E50-2440) BD Bioscience 1:1000
CD11b (M1/70), eBioscience 1:400
CD11c (N418), eBioscience 1:400
CD19 (1D3), eBioscience 1:400
NK1.1 (Pk136), Biolegends 1:400
Ter119 (TER119), eBioscience 1:400
PD-1 (J43), eBioscience 1:400
TIGIT (1G9), BD Bioscience 1:400
CD107a/LAMP1 (1D4B), eBioscience 0.5ug/test(100ul)
CD117(2B8) BV711, BD Bioscience, cat no. 563160
IFNg (XMG1.2) PerCP-Cy5.5 eBioscience 1:1500 Cat no. 45-7311-82
IL4 (11B11) APC eBioscience 1:200 Cat no 17-7041-82
IL13 (eBio13a) AF488 eBioscience, 1:400, Cat. no. 53-7133-82
IL17 (eBio64DEC17), APC, 1:200, Cat. no. 17-7179-42

Validation

Antibody validation information is available for each of the listed antibodies on the relevant manufacturer's website.

Eukaryotic cell lines

Policy information about cell lines

Cell line source(s)

C57BL/6 derived B16-F10 melanoma cell line was purchased from American Type Culture Collection (ATCC), E0771 cell line was kindly gifted by Robin Anderson.

Authentication

No cell line authentication was performed. Low passage stocks were used.

Mycoplasma contamination

All cell lines were screened and negative for mycoplasma

Commonly misidentified lines
(See ICLAC register)

No commonly misidentified cell lines were used in the study.

Animals and other organisms

Policy information about studies involving animals; ARRIVE guidelines recommended for reporting animal research

Laboratory animals

Wild type: C57BL/6. Genetically modified (generated at Sanger Institute): Cd4-Cre;Cyp11a1fl/fl and Cyp11a1-mCherry reporter. All animals used in this study were 8-12 weeks old. Experimental and control groups comprised littermates or sex-matched male and female mice with an age range not exceeding four weeks. Housing conditions at the Wellcome Sanger Institute: Mice were maintained in a specific pathogen free unit on a 12hr light: 12hr dark cycle with lights off at 7:30pm and no twilight period. The ambient temperature was 21 ± 2oC and the humidity was 55 ± 10%. Mice were housed for phenotyping using a stocking density of 3-5 mice per cage (overall dimensions of caging: (L x W x H) 365 x 207 x 140mm, floor area 530cm2) in individually ventilated caging (Tecniplast Seal Safe1284L) receiving 60 air changes per hour. In addition to Aspen bedding substrate, standard environmental enrichment of two nestlets, a cardboard Fun Tunnel and three wooden chew blocks was provided. Mice were given water and diet ad libitum. Mice were fed on Mouse Breeders Diet (Lab Diets, 5021-3). Animals recruited to studies at ARES Medical Research Coulcil animal facility remained socially housed in individually ventilated cages, at ambient temperature and with cage enrichment. Animals of each genotype were randomly assigned to experimental groups, and where possible, technicians performing the experiment were blinded to experimental groups and treatments.

Wild animals

No wild animals were used in the study.

Field-collected samples

No field collected samples were used in the study.

Ethics oversight

The care and use of all mice in this study were in accordance with the UK Animals in Science Regulation Unit's Code of Practice for the Housing and Care of Animals Bred, Supplied or Used for Scientific Purposes, the Animals (Scientific Procedures) Act 1986

Amendment Regulations 2012. All procedures were performed under a UK Home Office Project license (PPL 80/2574 or PPL P8837835 or PPL P6B8058B0), which were reviewed and approved by the Medical Research Council Laboratory of Molecular Biology (PPL 80/2574 or PPL P8837835) and Sanger Institute (PPLP6B8058B0) Animal Welfare and Ethical Review Bodies (AWERB).

Note that full information on the approval of the study protocol must also be provided in the manuscript.

Flow Cytometry

Plots

Confirm that:

- [x] The axis labels state the marker and fluorochrome used (e.g. CD4-FITC).
- [x] The axis scales are clearly visible. Include numbers along axes only for bottom left plot of group (a 'group' is an analysis of identical markers).
- [x] All plots are contour plots with outliers or pseudocolor plots.
- [x] A numerical value for number of cells or percentage (with statistics) is provided.

Methodology

Sample preparation	Tumors were mechanically dissociated and digested in 1mg/ml collagenase D (Roche), 1mg/ml collagenase A (Roche) and 0.4mg/ml DNase I (Sigma) in IMDM media containing 10% FBS, at 37°C for 40 mins. EDTA was added to all samples to neutralize collagenase activity (final concentration 5mM) and digested tissues were passed through 70µm filters (Falcon). For flow cytometry, we followed eBioscience surface staining, intracellular cytotoplasmic protein staining (for cytokines) protocols. Briefly, single cell suspension was stained with Live/Dead Fixable Dead cell stain kit (Molecular Probes/ Thermo Fisher) and blocked by purified rat anti-mouse CD16/CD32 purchased from BD Bioscience and eBioscience. Surface staining was performed in flow cytometry staining buffer (eBioscience) or in PBS containing 3% FCS at 40C. For intracellular cytokine staining cells were fixed by eBioscience IC Fixation buffer and permeabililzed by eBioscience permeabilization buffer. Cells were stained in 1x permeabilization buffer with fluorescent dye-conjugated antibodies. After staining cells were washed with flow cytometry staining buffer (eBioscience) or 3% PBS-FCS.
Instrument	Fortessa flow cytometer (BD Biosciences).
Software	FACS DIVA software (BD Biosciences) and FlowJo software (TreeStar).
Cell population abundance	>98% purity routinely achieved. Determined by flow cytometry (Fortessa) of post-sorted cells.
Gating strategy	The cells were first gated on the cell population on the FSC/SSC plot, termed as "all cells". Singlets were gated on FSC-A/FSC-W or FSC-A/FSC-H plot. Thereafter live cells were gated based on LIVE/DEAD Fixable Violet staining (Thermofisher). Afterwards, gated on CD45+ cells to gate immune cells. Then subsequent gating was based on the expression of surface and intracellular proteins.

- [x] Tick this box to confirm that a figure exemplifying the gating strategy is provided in the Supplementary Information.
