# Supplementary material for: Tumors induce de novo steroid biosynthesis in T cells to evade immunity
Source: Nat Commun. 2020 Jul 17;11:3588. doi: 10.1038/s41467-020-17339-6 (PMC7368057; doi:10.1038/s41467-020-17339-6)
Supplement: Supplementary file 2 — Peer Review File [file 41467_2020_17339_MOESM2_ESM.pdf]

Editorial note: This manuscript has been previously reviewed at another journal that is not operating a transparent peer review scheme. The manuscript was considered suitable for publication without further review at Nature Communications.
